# Applying EDTA in Chelating Excess Metal Ions to Improve Downstream DNA Recovery from Mine Tailings for Long-Read Amplicon Sequencing of Acidophilic Fungi Communities

**DOI:** 10.3390/jof8050419

**Published:** 2022-04-20

**Authors:** Rosina Nkuna, Grace N. Ijoma, Tonderayi S. Matambo

**Affiliations:** Institute for the Development of Energy for African Sustainability, University of South Africa, Christiaan De Wet/Pioneer Dr. P.O. Box X6, Florida 1710, South Africa; rosinamakofane@gmail.com (R.N.); nkechiijoma@gmail.com (G.N.I.)

**Keywords:** bioleaching, DNA extraction, ethylene diamine tetraacetic acid (EDTA), fungi, mine tailing

## Abstract

The hostile environment of mine tailings contains unique microbial life capable of bioleaching. The metagenomic analysis of such an environment provides an in-depth understanding of the microbial life and its potential, especially in biomining operations. However, DNA recovery from samples collected in those environments is challenging due to the presence of metal ions that interfere with the DNA analysis. A varied concentration of EDTA (4–13 µg/µL) to chelate the metal ions of enriched tailing samples prior to DNA extraction was performed. The results show that 9 µg/µL of EDTA was effective in most samples. However, the increasing concentration of EDTA negatively affected the DNA recovery. The sequencing of the successfully extracted DNA revealed a diverse range of fungal genera, some of which have not been previously reported in tailing or bioleaching applications. The dominant genera include *Fodinomyces*, *Penicillium*, *Recurvomuces*, *Trichoderma*, and *Xenoacremonium*; their traits were determined using the FungalTraits database. This study demonstrates the need to include a preliminary metal-chelating step using EDTA before DNA extractions for samples collected from metal-rich environments. It further showed the need for optimization but provided a benchmark range, particularly for tailings. However, we caution that a further EDTA removal step from the extracted DNA should be included to avoid its interferences in downstream applications.

## 1. Introduction

Mine tailings sites frequently contain distinct and phylogenetically diverse microbial communities that can be exploited for their specific metal-extraction capabilities (bioleaching) [1]. Despite the presence of both chemolithotrophic prokaryotes and heterotroph eukaryotes in these environments, the research is focused more on the chemolithotrophic members of the community in comparison to the eukaryotic members [2,3]. However, in recent years, the characteristics of the eukaryotic members, particularly their acid-tolerance, have piqued the interest of scholars and scientists in studying and applying them in bioleaching processes [4]. Depending on the research question, bioleaching research employs either a culture-dependent method or a culture-independent method or a combination of the two [5]. However, profiling such microbial communities relies on metagenomics approaches as they have been shown to be very useful in rapidly elucidating large sample areas. Metagenomic DNA isolated from tailing environments is a potential genetic resource from which the uncultured microbial species’ phylogenetic affiliation can be determined, and their genetic potential can be explored by identifying novel genes with potential applications in bioleaching processes [6].

The foundation of various biological applications in microbial profiling depends on accurate and reliable DNA analysis. Separating pure DNA from cell matrix or biological samples has proven to be extremely complicated and challenging in a complex environment [7]. Tailings are one of the most challenging environmental samples to recover pure DNA from [6]. They have high concentrations of heavy metals, which are impurities in the form of metal ions that have an impact on downstream DNA applications [8,9]. According to published research, these metal ions can interfere with the DNA analysis at various stages, from extraction to polymerase chain reaction (PCR) [6,10,11]. 

Metal ions cause interferences during DNA analysis; they act as cofactors required to increase the activity of various enzymes/nucleases such as Deoxyribonuclease (DNase) [12]. DNase is an endonuclease that hydrolyses double-stranded DNA, and divalent metal ions such as magnesium (Mg^2+^) and calcium (Ca^2+^) increase its activity [12,13,14,15]. Although DNA is packaged in a nucleus and protected from nucleases, it is exposed to nucleases during DNA extraction [16]. Metal ions in extracted DNA can also prevent successful PCR [10]; Ca^2+^, for instance, is an inorganic substance known to have inhibitory effects on PCR [13].

Commercial extraction kits/protocols are designed to extract DNA in its purest form, removing most impurities derived from the biological sample content or cell matrix [17]. To address metal ion removal or stability during extraction, lysis buffer contains chelating agents that are capable of stabilizing metal ions such as iron, magnesium, manganese, cobalt, zinc, lead, copper, calcium, etc. [10,18,19]. Chelating agents include ethylene diamine tetraacetic acid (EDTA) and ethylene glycol-bis(2-aminoethylether)-N,N,N′,N′-tetraacetic acid (EGTA) [10,17]. EDTA and EGTA have similar properties and can chelate nearly all the same metals, such as Mg^2+^, Cu^2+^, Fe^2+^, Mn^2+^, Ni^2+^, and Zn^2+^ ions [20]. However, the affinity of EDTA and EGTA for magnesium varies, with EDTA having a high affinity and EGTA having a low affinity [21]. EDTA chelates metal ions at a 1:1 ratio regardless of cation charge and forms stable chelating complexes with cations [22]. 

The metal content of environmental samples and the success of DNA extraction protocols differ depending on the sample’s chemical composition. According to Kuffel et al. [10], the impact of metal ion interference on the recovery of DNA is determined by the concentration of the various metals involved. As a result, the challenges of successful DNA extraction or PCR are expected with tailing samples with their associated high concentration of metal ions.

Nearly all of the previous investigations relating to metal interference in DNA extraction can only be found in forensic science, with studies conducted on methods of optimizing DNA extraction and PCR processes in relation to metal ion interferences and on blood, bone, and other samples collected from crime scenes [10,11,23]. Their findings thus far suggest the pre-treatment of samples containing significantly higher concentrations of metal ions with chelating agents such as EDTA, EGTA, and CHELEX (a chelating agent manufactured by Bio-Rad laboratories) before extraction [24]. This pre-treatment is an accepted protocol addition despite the included presence of chelating agents in all DNA extraction buffers found in commercial kits. However, it appears that the studies carried out investigating this likely interference for environmental samples, especially tailings, are few to non-existent

EDTA was chosen as the chelating agent in this study because it has a higher affinity for chelating Mg^2+^ ions (cofactor for DNase) compared to EGTA and does not interfere with most chemicals used in standard buffers. Moreover, previous studies demonstrated that the chelating ability of EDTA is effective between 4–13 µg/µL [23]. Beyond this concentration, EDTA may affect DNA recovery negatively. In this study, EDTA (4–13 µg/µL) was used as a metal ion chelating agent in tailing samples to optimize DNA recovery and PCR, with the goal of profiling the potential bioleaching fungal communities present in the tailing samples.

## 2. Materials and Methods

### 2.1. Site Description and Soil Sampling

Mine tailing samples were collected at three different tailing storage sites in Krugersdorp, Gauteng province, South Africa (mixed tailing and tailing A—26.132750, 27.711464, tailing B—26.133074, 27.767206, and tailing C—26.135979, 27.740172). A diagonal sampling method was used [25], and the samples were collected at various depths: depth 1: 0–15 cm, depth 2: 15–30 cm, and depth 3: 30–45 cm, while mixed tailings (mixed tailings before re-processing) were collected on the surface. Each depth’s samples were combined (for instance, all 0–15 cm depth samples were combined to form one sample per sampling site), and from each tailing storage site, a total of three samples were obtained. All samples were collected using sterile zipper bags and transported to the university laboratory on ice, and upon arrival, the samples were stored at 4 °C until further analysis.

### 2.2. Characterization of Mine Tailing Samples

The pH of the tailings was determined by dissolving 1 g of tailings in 10 mL of dH_2_O and allowing it to sit for an hour before taking pH measurements with an AD1030 pH meter. For heavy metal content analysis, 0.25 g samples were digested by mixing nitric acid (HNO_3_) and hydrochloric acid (HCL) at a ratio of 3:1 in a microwave digester (CEM Mars-6using Xpress plus vessels, MAD TECHNOLOGY (PTY) LTD, Johannesburg, South Africa), heated to 180 °C, and held there for 10 min. The samples were then diluted up to 50 mL in falcon tubes. The metals in the solution were initially scanned, followed by quantification using inductively coupled plasma atomic emission spectroscopy (ICP-AES) (Shimadzu ICPE-9820, Gauteng, South Africa).

### 2.3. Microbial Profiling and Isolation 

#### 2.3.1. Enrichment and DNA Extraction 

Two different selective media (Table 1) were used to enrich the fungal communities found in the tailing samples. The purpose of enrichment was to target the acidophilic/acid-tolerant communities with bioleaching potential and to increase their population for isolation. The two media were prepared as follows:

- Solution A contains all the compounds listed in Table 1 (for each media) except iron (II) sulfate or sulfur, which were sterilized by autoclaving at 112 °C for 30 min.
- Solution B contains either iron (II) sulfate (44.2 g/L in liquid media and 6 g/L on solid media, maintained at pH 2 to avoid oxidation) or sulfur (30 g/L in liquid media and 10 g/L) [26,27]. The solutions were sterilized by filtration using 0.2 µm sterile filters and added to solution A aseptically to prepare iron- or sulfur-containing media [28].

For the initial enrichment, only solution A was prepared, in which 20 g of tailings was added to 250 mL of the two specific media in Duran Schott bottles. The contents of the tailings were used as an energy source. The samples were enriched for 12 days at 30 °C in a shaker incubator at 140 rpm (Labotec (PTY) LTD, Model 353, Gauteng, South Africa); this was referred to as S1. After 12 days, 20 mL was transferred to the fresh liquid media containing both solution A and solution B (iron (II) sulfate heptahydrate served as the energy source) and incubated for a further 12 days; this was referred to as S2. 

#### 2.3.2. Optimization of DNA Extraction

Several failed attempts at extracting DNA and running a successful PCR from the enriched tailing samples led to the assumption that metal ions may have been interfering with the DNA extraction process. To stabilize the metal ions in the samples, 10 mL of S1 and S2 samples was centrifuged at 14,000 rpm, and the supernatant was discarded. The pellet was resuspended with EDTA (4–13 µg/µL) and treated at room temperature for an hour on a rotating mixer at 60 rpm before extraction [24]. The samples were further centrifuged to remove the EDTA and resuspended in 250 µL dH_2_O. The samples with no EDTA treatment were centrifuged and only resuspended in dH_2_O. DNA extractions were conducted using Zymo soil/faecal and BIOMICS extraction kits (Inqaba Biotechnical Industries (Pty) Ltd., Pretoria, South Africa), according to the manufacturer’s instructions. The ratio of absorbance at 260 and 280 nm was used to check the purity of the extracted DNA using a nanodrop (Mfg year 2016) and quantified using a QUBIT fluorometer (ThermoFisher, Edenvale, South Africa). To further confirm the chelating ability of EDTA, a PCR amplification of the ITS gene was conducted using ITS1—5′ CTTGGTCATTTAGAGGAAGTAA-3′ and ITS4—5′ CCTCCGCTTATTGATATGC-3′ primers [31]. The ITS gene was amplified in a 25 µL reaction containing DNA template, 5 µM of each primer, and 12.5 µL of Biolabs 2X Master mix (Inqaba Biotechnical Industries (Pty) Ltd., Pretoria, South Africa). The PCR amplification was performed as follows: initial denaturation at 95 °C for 30 s, 35 cycles of denaturation at 95 °C for 30 s, annealing at 55 °C for 1 min, extension at 68 °C for 1 min, and a final extension at 68 °C for 5 min. One percent agarose gel electrophoresis was used to view the amplified gene. The DNA samples treated with EDTA that resulted in a successful PCR amplification were submitted to Inqaba Biotechnical Industries (Pty) Ltd., Pretoria, South Africa for long amplicons sequencing with PacBio sequel II.

#### 2.3.3. Bioinformatics and Statistics 

A total of 1,375,847 demultiplexed reads were obtained across 46 samples, with an average of 29,909 reads per sample. The DADA2 ITS pipeline workflow [32] with technology specific for PacBio [33] was used. The DADA2 R package v1.18.0 was installed on R studio. The sequences were quality filtered and denoised, and the chimeric sequences were removed. The DADA2 default naïve Bayesian classifier [34] was used to assign taxonomy to the amplicon sequence variants based on the UNITE database [35]. The resulting OTU table with assigned taxonomy was exported to Excel for relative abundance plots at different levels. The alpha and beta diversities plots were generated using MicrobiomeAnalyst (a comprehensive statistical, visual, and meta-analysis of microbiome data).

## 3. Results

### 3.1. Tailing Characterization 

**Figure 1:**
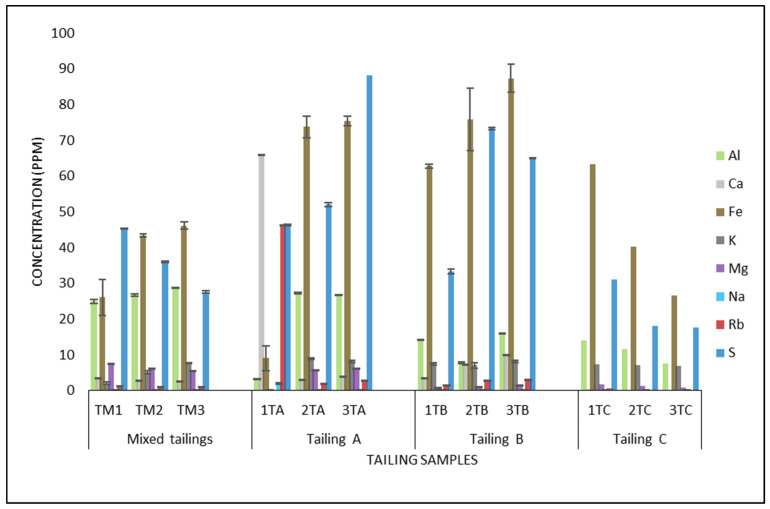
Heavy metal characterization of 4 different tailings at different depths. 1–3—sampling depth, T—tailing, A–C—different tailings, and M—mixed tailing.

Table 2 shows the pH measurements of the four different tailing samples, which ranged from 3.9 to 1.8 in all four tailing sites. The pH range in tailings A and B is the lowest. Figure 1 depicts the metal content analysis of the tailings at various depths using ICP-EOS. The highest concentrations of iron and sulfur were observed in all the tailings, followed by aluminum in varying concentrations. The differences in the tailings revealed that tailings A and B had the highest concentrations of the three metals, with iron at 75.7 ppm and 87.3 ppm and sulfur at 88.1 ppm and 73.2 ppm, respectively. Rubidium and calcium were observed to be highest in tailing A only at 46.3 and 65.8 ppm, respectively. Rubidium (except in tailing A depth 1), Calcium (except in tailing A depth 1), potassium, and sodium were found in lower concentrations.

### 3.2. DNA Optimization 

An EDTA concentration of 9 µg/µL was found to be effective for metal ion chelation in S1 samples. This was confirmed by comparing the 260/280 ratio DNA concentration and the successful PCR amplification of the ITS1–ITS4 region in S1 samples from both enrichment media. The 260/280 ratio and the DNA concentration improved in the EDTA-treated samples (Table 3). The average ratio of the samples without EDTA treatment in GYEM and YSM was 1.68 and 1.18, respectively, and improved to 2.0 and 1.75, respectively, in the samples treated with EDTA. The DNA concentration increased from 1.31 ng/mL in GYEM and 0.35 ng/mL in YSM in the control samples to 74.4 ng/mL and 30.7 ng/mL, respectively, in the EDTA-treated samples. The PCR amplification of the ITS region was successful in all the EDTA-treated DNA samples (Figure S1). 

EDTA concentrations ranging from 9–13 µg/µL were used in the S2 samples. The average ratio of the samples without EDTA treatment in GYEM and YSM was 1.72 and 1.07, respectively, whereas the average ratio improvement in the samples treated with EDTA was 2.38 and 1.70, respectively. In GYEM, the average DNA concentration increased from 1.52 ng/mL in the samples that were not treated with EDTA to 39.72 ng/mL in the samples that were treated with EDTA. In YSM S2, the samples without EDTA treatment yielded no DNA, whereas the samples with EDTA yielded 1.92 ng/mL on average. Amplification of the ITS region by PCR was mostly successful in the GYEM S2 samples treated with EDTA (Figure S1).

### 3.3. Alpha and Beta Diversities 

**Figure 2:**
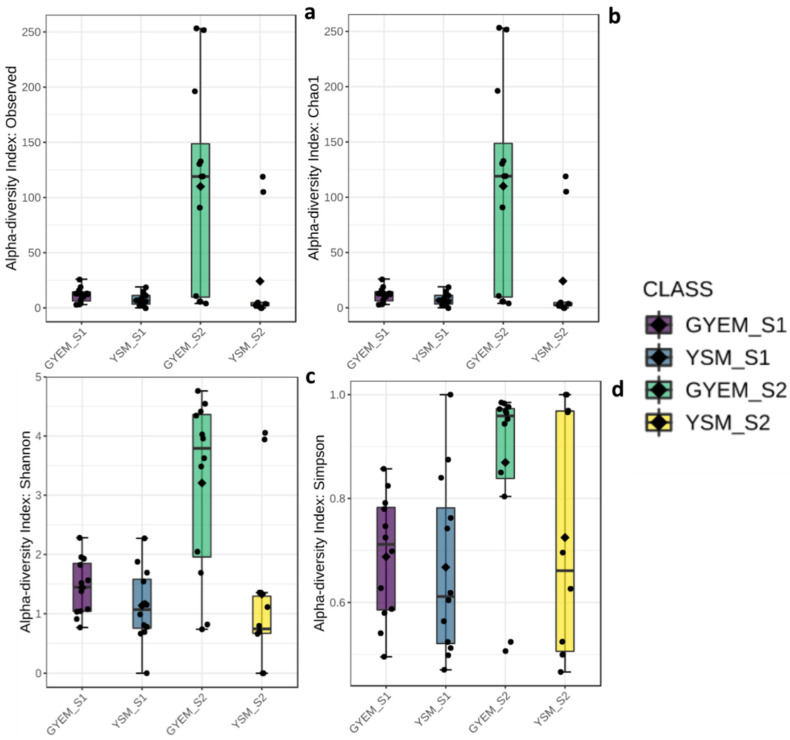
Alpha diversity indices of fungi: (**a**)—observed OTUs (richness index), (**b**)—Chao1 (total richness), (**c**)—Shannon index (richness and evenness) and (**d**)—Simpson index (number of species present, as well as the relative abundance of each species).

**Figure 3:**
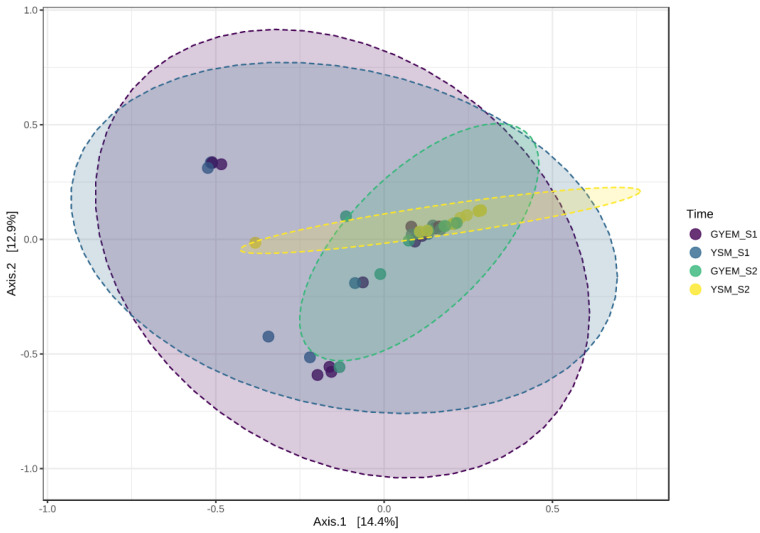
Bray−Curtis measures of beta-diversity visualised using principal coordinate analysis (PCoA) for the comparison of fungal diversity for enrichment (S1 and S2) of tailing samples using GYEM and YSM.

A total of 766,039 sequences were clustered into 564 OTUs, after filtering, using the following criteria—a minimum count of 4 OTUs per sample and the OTU number was 167 using MicrobiomeAnalyst (a comprehensive statistical, visual, and meta-analysis of microbiome data). The alpha diversities were measured at the feature level using the Kruskal–Wallis statistical method (Figure 2); significant differences for alpha-diversity indices S1 and S2 were observed. The statistical p-values were as follows: observed and chao1—*p* < 0.002, Shannon—*p* < 0.003 and Simpson—*p* > 0.006. The highest diversity index was observed in GYEM S2, and the lowest diversity index was observed in YSM S2. The beta diversity was also measured using the Bray–Curtis index distance method, and the PERMANOVA statistical method was used (F-value: 2.5639; R-squared: 0.15479; *p*-value < 0.001) (Figure 3). Beta diversity compares the number of species shared between samples; from Figure 3, overlaps can be seen from all the four grouped samples (based on media (GYEM and YSM) and enrichment (S1 and S2).

### 3.4. Taxonomic Diversity of Microbial Community on GYEM and YSM Enrichment

**Figure 4:**
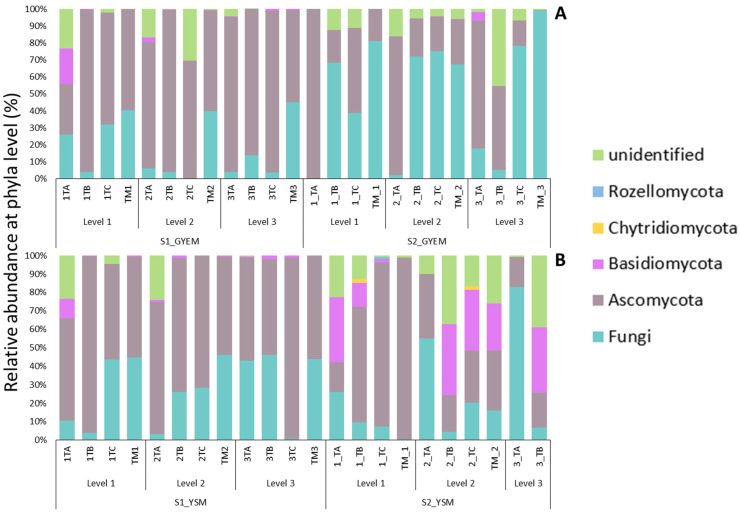
Relative abundance of fungi at phyla depth: (**A**)—glucose yeast extract media and (**B**)—yeast sucrose media.

**Figure 5:**
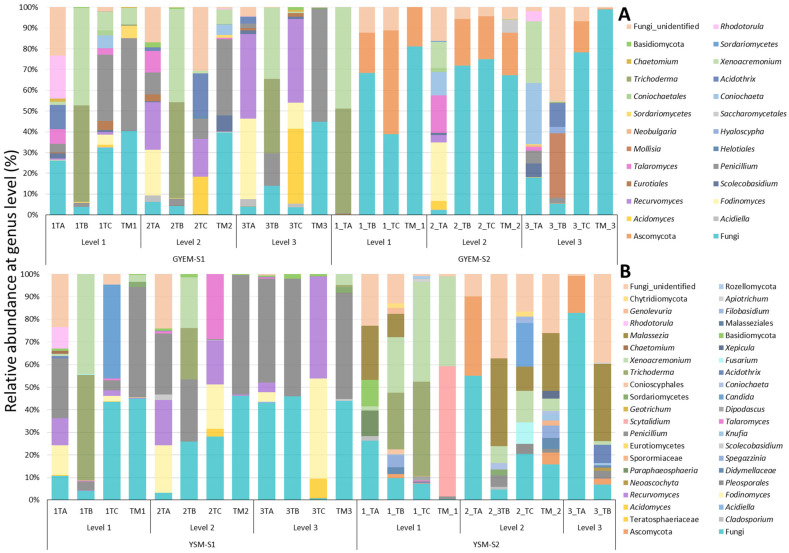
Relative abundance of fungi at genus depth: (**A**)—glucose yeast extract media and (**B**)—yeast sucrose media.

The relative abundance of the microbial communities enriched with glucose yeast extract and yeast sucrose media was determined at the phylum and genus depths comprising at least 1% in at least one sample (Figure 2 and Figure 3). There was an abundance of the Ascomycota, Basidiomycota, Chytridiomycota, and Rozellomycota phyla (Figure 4). Despite the apparent dominance of these phyla in the various tailing sites, each enrichment medium had its own distinct fungal community composition (Figure 5). The phylum Ascomycota was the most prevalent in both enrichment media, represented by the genera *Penicillium*, *Xenoacremonium*, *Talaromyces*, *Fodinomyces*, *Recurvomyces*, and *Trichoderma*. The phylum Basidiomycota was abundant in YSM-S2, with the genera Rhodotorula and Malassezia representing this phylum. 

The enrichment media were selected to target the acid-tolerant fungi with bioleaching potential. The microorganisms in the tailings were first enriched in GYEM and YSM using the content of the tailing as a source of energy (S1) and then sub-cultured in the same media, but iron (II) sulfate heptahydrate was used as a source of energy (S2). The following paragraphs compare the diversity abundance of S1 and S2 on both enrichment media per tailing site at genus level (Figure 5). Furthermore, by using the PacBio platform to sequence the full-length ITS region of the extracted DNA, the taxonomic profile of some fungal populations in the tailing samples was resolved to the species level (see Figure S2).

Tailing A: The genera *Penicillium*, *Fodinimyces*, and *Recurvomyces* were observed in most depths of the tailing A samples for both media in the S1 and S2 enrichments, ranging from 4.2–45%. *Talaromyces* was observed only in GYES-S1 depth 1 and 2 (7.09% and 10.5%), while Trichoderma and *Xenoacremonium* were observed only in depth 1 and 2 of GYEM-S2 (12.6–50.6%). The genus *Rhodotorula* was only observed in depth 1 S1 of both media enrichment but was most dominant in GYEM-S1 at 20.6%. Unique genera to GYEM-S2 depth 1 and 3 were Trichoderma (50.6%), *Scoleobasidium* (6.6%), and *Acidothrix* (20.8%), whereas Paraphaesophaeria (11.3%) *Basidiomycota* (11.5%), and *Malassezia* (23.8%) were unique to YSM-S2 depth 1.

Tailing B: Similar to tailing A, *Penicillium*, *Fodinomyces*, and *Recurvomyces* were observed in most depths of the tailing B samples for both media in the S1 and S2 enrichments, ranging from 15.7–52%. *Malassezia* was only observed in YSM-S2 depth 1 and 2 at 10.11% and 38.4%, respectively, while Acidothrix was observed in S2 depth 3 in both media. *Mollisia* (3.1%) was unique to GYEM-S2 depth 3, and *Spegazzinia* (5.3%) was unique to YSM-S2.

Tailing C: The genera *Penicillium*, *Fodinimyces*, *Acidomyces*, and *Recurvomyces* were observed in most depths of the tailing C samples for both media in S1 and S2 enrichments, ranging from 4.9–44%. *Penicillium* was observed in S1 of both media (GYEM-S1 depth 1 at 31.9% and 2 at 9.7%, YSM-S1 depth 1 at 4.1%). *Xenoacremonium* (8.9%) was observed in the GYEM-S1 depth, while in YSM-S2 it was observed at depth 1 and 2 (43.6% and 13.8%). *Talaromyces* (28.7%), *Coniochaeta* (6.2%), and *Acidothrix* (20.3%) were unique to GYEM-S1 depth 1 and 2, while *Candida* (41.8%) was unique to YSM-S2 depth 2 and *Fusarium* (9.5%) and Pleosporale (4.4%) were unique to YSM-S2 depth 2.

Mixed tailings: The genera *Penicillium* and *Xenoacremonium* were abundant in most depths of mixed tailing samples for both media in the S1 and S2 enrichments, ranging from 7.9–52.7%. Sorduriomycetes was observed in both media S1 but was most abundant in GYEM at 5.87%. *Scolecobasidium* (7.5%) was unique to GYEM-S1 depth 2, whereas *Scytalidium* (57.6%), *Malassezia* (25.6%), and Spegazzinia (5.3%) were unique to YSM-S2 depth 1 and 2.

The FungalTraits database was used to determine the dominant fungal taxa’s ecological information and functional assignment [36,37]. This database categorizes the functional assignment into various “traits”, such as primary and secondary lifestyles, endophytic interaction capacity, aquatic habitat, and many others. The FungalTraits database revealed ecological and functional information for eight of the 17 dominant genera. According to the findings of the ecological functional assignment, for primary lifestyle, the genera are mostly soil and wood saprotrophs. Their secondary lifestyle and endophytic interaction capacity are primarily foliar-endophytic, with a few genera assigned to root-associated and rock-inhabiting. Table 4 contains more detailed information on the ecological data and the functional assignment found for the fungal isolates present in this study.

## 4. Discussion

### 4.1. DNA Extraction Optimization 

Metal ion interference on DNA recovery is largely determined by metal concentration [10]. As shown in S1 and S2 of both enrichment media, the effectiveness of EDTA in chelating metal ions varies with the metal content and concentration. In S1, the main energy source was the tailing contents (Figure 1), whereas in S2 the main energy source was the inclusion of iron (II) sulfate heptahydrate. As a result of the higher Fe^2+^ ion concentrations, the S2 samples may have required a higher EDTA concentration to complete chelation. Fe^2+^ may also be responsible for the low DNA recovery and the encountered difficulty in running PCR in S2 samples. These findings are similar to an observation made by Kuffel et al. [10] when they investigated metal ion interferences in forensic DNA analysis from crime scenes, where DNA extractions were carried out from swabs of a variety of metal objects, including bullets, cartridge casings, gun surfaces, knives, metal wires, and surfaces, as well as calcium-rich bone samples. They caution that the metal ions’ impacts on DNA recovery, extraction, and amplification are not entirely understood. However, their investigations demonstrate the effects of some metals on DNA amplification, showing that inhibitory effects were observed with zinc, tin, iron (II), and copper, with 50% of the inhibitory concentration (IC50) values much below 1 mM. Thus, their study finding supports the observation made in this present study regarding the inhibitory effects observed with Fe^2+^ ions. 

The low recovery of DNA and failed PCR may also be attributed to the high concentration of EDTA used in S2 as compared to the S1 samples. According to a study conducted by Khosravinia and Ramesha [23], EDTA concentrations > 11 µg/µL are known to reduce DNA recovery. Beyond chelating, Lopata et al. [20] also showed that EDTA affects enzyme activity by tightly binding to the active site of enzymes such as Taq DNA polymerase, dUTPase, and dNTPase, resulting in low or no PCR products. As an example, Taq polymerase requires Mg^2+^ for activation, while EDTA tends to compete with Mg^2+^ for binding, resulting in enzyme inhibition. This trend of the poor activity of enzymes in the presence of higher concentrations of EDTA and metal ions was also observed by Kuffel et al. [10] and they highlighted that KOD polymerase was more metal-resistant than Taq polymerase in such instances

A ratio of absorbance at 260 and 280 nm can be used to assess DNA purity, and a ratio of ≈1.8 and even up to 2.0 is regarded as pure. However, a ratio above 2.0 shows the likelihood of RNA contamination of the DNA being assessed for purity. Moreover, a ratio of >2 is related to DNA degradation and is showing, rather, a measurement of free nucleotides, as described in the protocol manual for the NanoDrop instrument [38]. Moreover, a ratio of 1.6 suggests the presence of proteins, phenol, or other contaminants that absorb strongly at or near 280 nm [39]. The S2 samples had an average ratio of 1.7 after treatment with EDTA, which is likely due to any one of the factors described.

### 4.2. Taxonomic Diversity of Microbial Community in Tailing Samples

Metamorphic environments such as tailings are characterized by high metal concentrations and extremely low pH ranges. These factors contribute to the diversity structure of the acidophilic or acid-tolerant communities found in that environment [40,41]. As a result, the taxonomic abundance of the fungal community observed in the four tailing samples was influenced by their habitat. Furthermore, the nutrients provided by the enrichment media also contributed to the taxonomic abundance. Tailing A and B had the lowest pH ranges of <2.1, which decreased with each depth, whereas mixed tailing and tailing C had pH ranges of >3 for all depths. However, the four tailings had similar metal content with high concentrations of iron, sulfur, and aluminum, though the concentration differed (Figure 1). This explains why genera such *Penicillium*, *Fodinomyces*, and *Recurvomyces* were dominant in most depths of the different tailings. *Penicillium* and *Fodinomyces* are both acidophilic (growing only at pH below 5) and acid-tolerant (growing at pH ranging below or above 5) [1]. Furthermore, their abundance in both enrichment media demonstrates their ability to thrive in different carbon sources [1,42]. *Penicillium* and *Fodinomyces* have been applied in bioleaching and have demonstrated metal tolerance and good bioleaching efficiencies [1,43]. In our research, no report of *Recurcomyces* in tailing samples or its application in bioleaching was found, making this report the first report of its identification in tailings. However, this genus has been described by Selbmann et al. [44] as a rock-inhabiting yeast found in cold, dry environments, and this ecological feature is supported by analysis conducted using the FungalTraits database (Table 4). Furthermore, Coleine et al. [45] suggested that nonsporulating fungi found in rock habitats may occasionally be dispersed between continents, for example via dust, and adapt to other ecologically similar habitats. As a result, the characteristics of *Recurvomyces*, such as growing in an extreme environment, as described by Coleine et al. [45], Selbmann et al. [5], Ametrano et al. [46], and Selbmann et al. [44] are evident enough to support the possibility of its existence in tailings. Further investigations of this genus’ bioleaching efficiency are therefore required.

Another dominant genus that was observed in most of the tailings (except in tailing B), especially in depths 1 and 2, is *Xenoacremonium*. This genus, previously referred to as *Acremonium recifei* (now renamed *Xenoacremonium recifei* L. by Lombard and Crous) [47], is a plant pathogen involved in wood decay and has also been isolated from petroleum-contaminated sites [48,49]. Ecological information from FungalTrails also shows that it is a wood saprotroph; thus, its characterization as a wood decaying pathogen is valid. This is likely the first report of this genus in tailing samples. No studies have yet linked this genus to bioleaching processes or applications. *Acidothrix* is also another genus observed to be dominant mostly in depth 3 of the tailing samples (except tailing C). *Acidothrix* exhibits both acid-tolerant and acidophilic characteristics and is mostly found in extreme habitats such as acidic soils [50]. This explains its detection only at depth 3, where the lowest pH ranges were observed. 

Although similar genera were observed in most tailings, some genera were only found in specific tailing samples. For example, *Trichoderma*, *Rhodotorula*, and *Paraphaesophaeria* were only observed in tailing A, *Mollisia* in tailing B, *Candida*, *Acidomyces*, *Coniochaeta*, *Fusarium*, and *Pleosporale* in tailing C, and *Scytalidium* in mixed tailings. Though they are unique to each tailing, *Trichoderma*, *Candida*, *Acidomyces*, *Rhodotorula*, *Fusarium*, *Coniochaeta*, and *Scytalidium* have been observed to share similar characteristics such as acid and metal tolerance. Their occurrence in tailing samples has been reported as well as their application to the bioleaching process [43,51,52]. The fungus *Paraphaesophaeria* was only found in tailing A and is known to be a plant-associated (primarily a desert plant) fungi with Cu, Cr, and Cd tolerance [53]. It has also been reported in manganese oxidation [54]. *Mollisia* is another genus found only in tailing B. It is an endophyte that is associated with plants and is also involved in plant decay [55,56]. Its presence in tailings is likely due to the current efforts to revegetate tailings to prevent dust dispersion during windy days, as well as other environmental concerns linked to airborne pollution [57]. 

A comparison of the observed genera in the two media revealed genera that were unique to each. For example, *Malassezia* and *Spegazzinia* were observed only in YSM S2 and *Scoleobasidium*, which was unique to GYEM. The genus *Malassezia* has been reported in flotation tailings of the Kevitsa mine in Finland [58] and in zinc and copper tailings [59]. Because the genus is known to dominate parts of the human microbiota [60], it was suggested by Miettinen et al. [59] that its presence in tailing samples may have come as result of human contamination. However, ecological information from FungalTraits assigned *Malassezia* as a soil saprotroph that is associated with plant roots. This may be a demonstration of the genera’s versatility and mobility. Thus, its ability to survive in low pH and high concentrations of iron (II) sulfate heptahydrate in the S2 media enrichment, as observed in this study, implies that members of this genus are potentially acid-tolerant and further investigations may be required to determine their bioleaching potential. According to Amend [61], *Malassezia* are ecologically hyper-diverse and have been reported in diverse environmental sources, such as Antarctic soils [62], hydrothermal vents, and deep-sea sediments [61]. *Spegazzenia* is a wood saprotroph with no endophytic capability, as described in Table 4, obtained from FungalTraits. This genus of *Spegazzenia* was observed by Samarakoon et al. [63] to be associated with *Musa* sp. (banana). No reports of *Spegazzenia* in the past have associated any member of this genus with tailing or bioleaching. *Scoleobasidium* is an endophyte that was previously isolated in tomato fruits, as reported by Mahmoud and Narisawa, [64]. The genus showed the ability to increase plant biomass in the presence of an organic nitrogen source. The plant growth promoting ability was also reported by Hamayun et al. [65] in soybean plants. Therefore, just like *Mollisia*, its introduction to tailings is likely due to the revegetation process at these tailing sites. These four genera, however, have not been previously reported in association with bioleaching processes and will require extensive investigations. This validates the need for routine bioprospecting of extreme environments for microorganisms that may potentially be useful for industrial applications and biomining operations.

## 5. Conclusions

In this work, the application of EDTA to stabilize divalent metal ions was explored as a result of the difficulties encountered in extracting DNA from the metal-rich environment of tailings. Our investigation showed that the use of EDTA improved the DNA recovery for S1 samples with no interference with the downstream application. However, in the S2 samples, the use of a high EDTA concentration led to low DNA recovery, which impacts the downstream application. It is therefore recommended that a necessary preliminary application of optimal concentrations of EDTA is required to extract DNA from environmental samples obtained from metal-rich environments such as those present in tailings. However, we caution that a further EDTA removal step must be included above the conventional wash step advised by the DNA extraction kit manufacturers. The elimination of the additional EDTA is needed to ensure the integrity of the DNA extracts and to prevent enzyme inhibition during downstream analysis. 

The fungal ITS1 long-read amplicon sequencing revealed the presence and varied compositions of the acidophilic and acid-tolerant fungal diversity at different depths of the four tailing samples. It further elucidated novel fungi genera that had not been previously identified in tailing samples and were likely unique to these mines in Krugersdorp, Gauteng, South Africa. The findings of this study clearly support the use of culture-independent techniques and long-read amplicon sequencing to gain a more in-depth understanding of biodiversity. However, culture-independent studies do not provide enough opportunities to visualize (morphological characteristics), to test functions (such as bioleaching ability), and to investigate the genetics and genomics of these acidophiles. This limitation is being solved by the creation of the FungalTraits database. Nonetheless, they provide great insights with their high throughput that prompt further investigation, particularly with regard to applications and further tests of microbial efficiencies and abilities. As such, they are excellent tools for bioprospecting vast sample areas.

## Figures and Tables

**Table 1:** Media composition for enrichment and isolation of bioleaching microorganisms.

Name	Composition		
**General**			
Basic bioleaching media composition	3.0 g/L (NH_4_)_2_SO_4_, 0.5 g/L MgSO_4_·7H_2_O, 0.5 g/L K_2_HPO_4_, 0.1 g/L KCl, 44.2 g/L FeSO_4_·7H_2_O or 30 g/L sulfur and 1.5% Agar bacteriological		
**Compounds Different in Each Media**		**pH**	**References**
Glucose yeast extract medium (GYEM)	5 g/L glucose, 0.05 g/L yeast extract	3	[29]
Yeast sucrose media (YSM)	100 g/L sucrose, 1.5 g/L NaNO_3_, 1.6 g/L yeast extract	3	[30]

**Table 2:** pH measurements of the different tailings.

Depths	Mixed Tailings	Tailing A	Tailing B	Tailing C
1: 0–15 cm	3.27 ± 0.75	2.1 ± 0.017	3.0 ± 1.06	3.945 ± 0.02
2: 15–30 cm	3.305 ± 0.46	1.8 ± 0.04	2.8 ± 1.12	3.7 ± 0.04
3: 30–45 cm	3.25 ± 0.61	1.8 ± 0.005	2.8 ± 1.12	3.7 ± 0.01

**Table 3:** Effectiveness of EDTA as chelating agent based on DNA purity, concentration, and successful amplification of ITS region.

Sample ID	A260/A280				Fluorometer (ng/mL)				Reads Count	
	Glucose Yeast Extract Media									
	S1		S2		S1		S2		S1	S2
	No EDTA	EDTA	No EDTA	EDTA	No EDTA	EDTA	No EDTA	EDTA	EDTA	EDTA
TM1	2.0	1.95	1.83	2.50	1.25	78.5	1.50	14.4	43,557	25,392
TM2	1.55	2.00	2.0	2.33	1.18	1.09	2.00	2.08	45,882	43,225
TM3	1.40	2.00	1.50	2.66	low	0.59	2.50	14.5	10,319	76,036
1TA	1.60	2.10	2.10	2.40	1.31	140	0.58	19.6	60,803	3868
2TA	1.5	2.00	1.93	2.70	0.98	172	1.95	7.59	19,980	6725
3TA	2.0	2.00	1.55	2.75	1.51	6.64	0.88	7.72	60,633	6365
1TB	1.66	1.92	2.00	2.20	0.89	225	2.80	42.1	34,189	125,058
2TB	1.50	1.9	1.01	2.20	low	37.8	0.94	7.61	51,553	44,624
3TB	2.0	1.95	1.35	2.20	0.84	139	1.21	13.5	43,365	22,567
1TC	1.71	2.14	1.88	2.25	1.29	8.88	low	2.09	37,915	45,172
2TC	1.50	2.14	1.6	1.92	1.07	26.0	3.08	340	31,218	100,441
3TC	1.80	2.00	2.00	2.50	5.48	58	0.86	5.54	7045	75,451
	**Yeast Sucrose Media**									
	**S1**		**S2**		**S1**		**S2**		**S1**	**S2**
	**No EDTA**	**EDTA**	**No EDTA**	**EDTA**	**No EDTA**	**EDTA**	**No EDTA**	**EDTA**	**EDTA**	**EDTA**
TM1	1.7	1.62	0.22	4.00	1.85	14.8	low	0.75	29,426	9131
TM2	1.54	1.50	1.40	1.50	1.47	1.54	low	0.88	27,541	681
TM3	1.62	1.88	1.35	1.50	0.98	5.74	low	1.32	38,058	failed
1TA	1.70	1.7	0.18	--	low	9.31	low	0.50	22,704	568
2TA	0.18	1.85	1.80	2.50	low	7.79	low	2.60	23,805	33,873
3TA	1.27	1.87	1.44	2.25	low	183	low	3.37	24,542	20,572
1TB	0.90	1.78	1.35	1.36	low	64.4	low	2.06	34,033	1193
2TB	1.44	1.62	1.55	1.58	low	10.1	low	3.78	6816	1433
3TB	0.71	1.72	1.28	2.00	low	16.5	low	2.57	11,643	1261
1TC	1.0	1.86	0.81	2.00	low	45.1	low	1.19	13,849	3809
2TC	1.40	1.93	0.23	--	low	9.34	low	2.81	20,693	1685
3TC	0.75	1.75	1.27	1.75	low	1.30	low	1.21	27,148	failed

Note: 1–3 represents depths (1: 0–15 cm, 2: 15–30 cm, 3: 20–45 cm), respectively. TM—mixed tailing; TA—tailing A; TB—tailing B, and TC—tailing C.

**Table 4:** Ecological information and functional assignment of dominant fungal taxa using FungalTraits database.

Genera	Primary Lifestyle	Secondary Lifestyle	Comment on Lifestyle	Endophytic Interaction Capability	Plant Pathogenic Capacity	Decay Substrate	Decay Type	Aquatic Habitat	Animal Biotrophic Capacity	Growth Form	Fruitbody Type
*Penicillium*	unspecified saprotroph	foliar endophyte	toxin-producing, animal parasite some species, mycoparasite some species	foliar endophyte	0	soil	mold	partly aquatic	opportunistic human parasite	filamentous mycelium	0
*Recurvomyces*	soil saprotroph	rock-inhabiting	0	0	0	soil	0	non-aquatic	0	filamentous mycelium	perithecium (hymenium hidden, narrow opening)
*Talaromyces*	unspecified saprotroph	0	hypervariable, thermophile, some species animal parasite some species various saprotrophs	0	0	leaf/fruit/seed, soil, animal material	mold	partly aquatic	opportunistic human parasite	filamentous mycelium	0
*Xenoacremonium*	wood saprotroph	0	0	0	0	wood	0	partly marine (partly non-aquatic)	opportunistic human parasite	filamentous mycelium	0
*Rhodotorula*	unspecified saprotroph	foliar endophyte	0	foliar endophyte	0	0	0	partly aquatic	opportunistic human parasite	yeast	none
*Acidothrix*	soil saprotroph	0	0	0	0	soil	0	non-aquatic	0	filamentous mycelium	none
*Malassezia*	soil saprotroph	root-associated	0	root-associated	root-associated	roots, soil	0	partly aquatic	animal-associated/opportunistic human parasite	yeast	none
*Spegazzinia*	wood saprotroph	0	0	no endophytic capacity	0	wood	0	non-aquatic	0	filamentous mycelium	perithecium (hymenium hidden, narrow opening)
*Coniochaeta*	unspecified saprotroph	foliar endophyte	hypervariable	foliar endophyte	leaf/fruit/seed pathogen	leaf/fruit/seed, soil, dung, animal material	0	non-aquatic	animal parasite	filamentous mycelium	cleistothecium (closed, spherical)

*Fodinimyces*, *Trichoderma*, *Scoleobasidium*, *Paraphaesophaeria*, *Mollisia*, *Sardariomycetes*, and *Scytalidium* were not detected on FungalTraits database.

## Data Availability

The original sequencing data obtained in this study were submitted to the NCBI Sequence Read Archive (SRA) database (fungi accession number: PRJNA803379).

## Supplementary Materials

The following supporting information can be downloaded at: https://www.mdpi.com/article/10.3390/jof8050419/s1, Figure S1: gel image showing samples with successful amplification of ITS region, Figure S2: evolutionary relationships of taxa resolved to species level.
