# Supplementary material for: Applying EDTA in Chelating Excess Metal Ions to Improve Downstream DNA Recovery from Mine Tailings for Long-Read Amplicon Sequencing of Acidophilic Fungi Communities
Source: J Fungi (Basel). 2022 Apr 20;8(5):419. doi: 10.3390/jof8050419 (PMC9143545; doi:10.3390/jof8050419)
Supplement: Supplementary file 1 [file jof-08-00419-s001.zip › jof-1640579-supplementary.pdf]

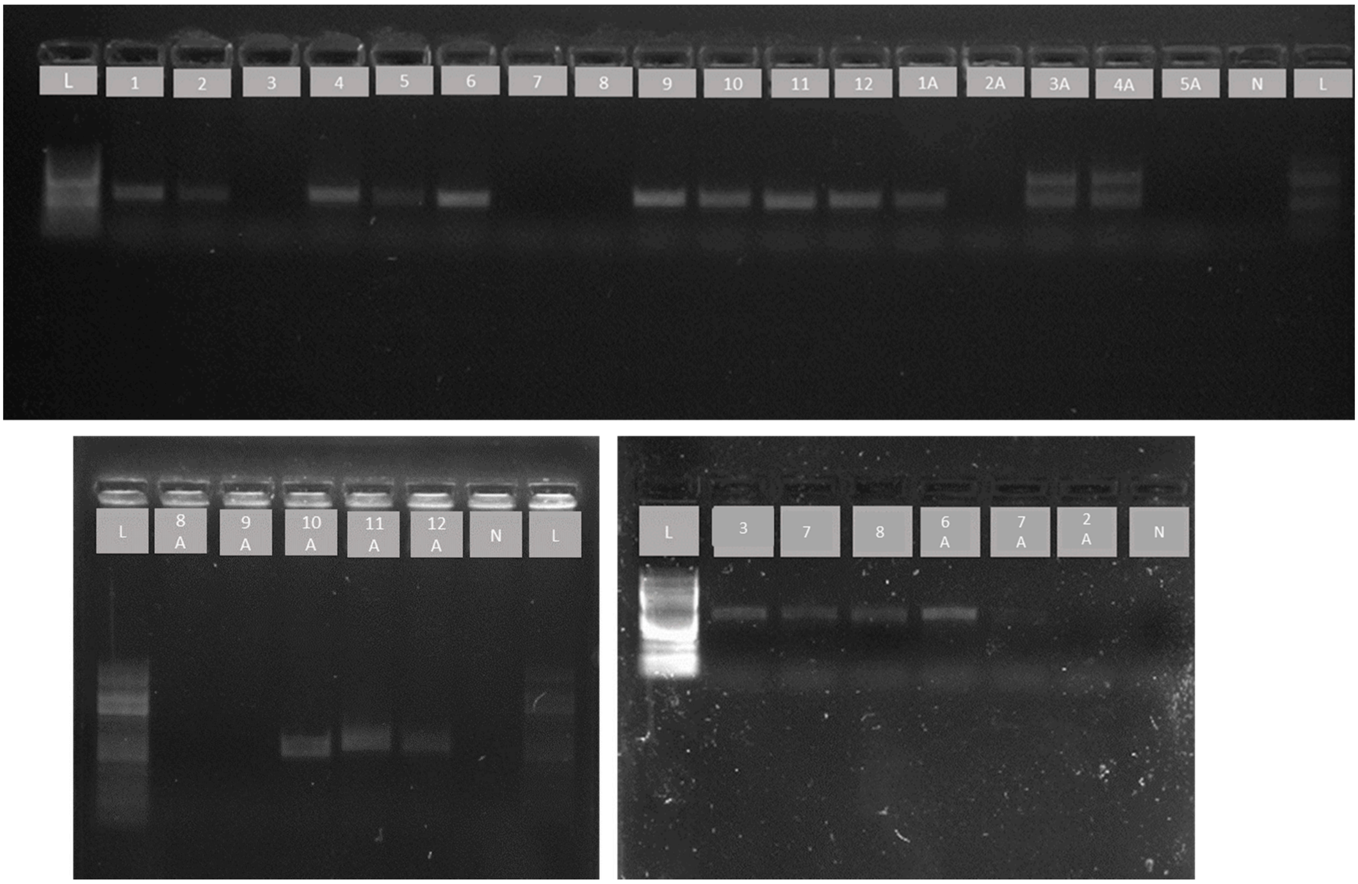

Figure S1. Gel image showing samples with successful amplification of ITS region. 1-10 represent TM1, TM2, TM3, 1TA, 2TA, 3TA, 1TB, 2TB, 3TB, 1TC, 2TC, 3TC for S1 respectively and 1A to 10A represent TM1, TM2, TM3, 1TA, 2TA, 3TA, 1TB, 2TB, 3TB, 1TC, 2TC, 3TC for S2 respectively. L-ladder and N -Negative.

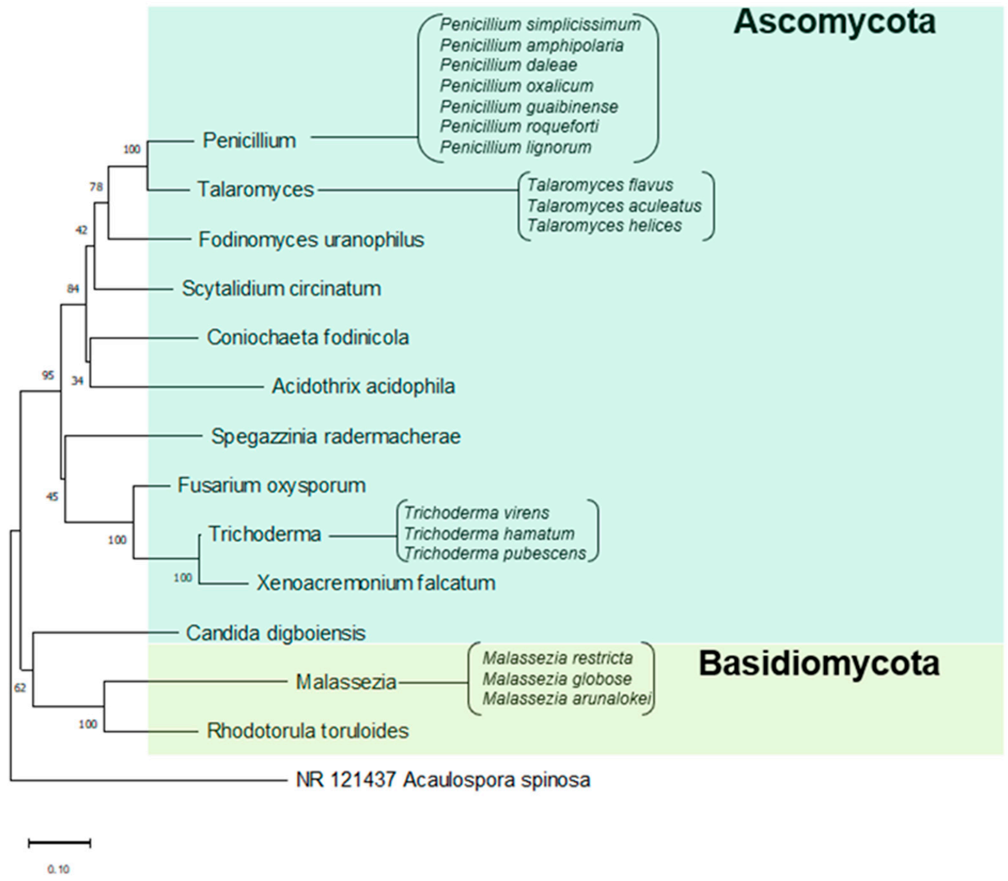

Figure S2. Evolutionary relationships of taxa resolved to species level. The evolutionary relationships of taxa are resolved to the species level. The Neighbor-Joining method was used to infer the evolutionary history [36]. Next to the branches is the percentage of replicate trees in which the associated taxa clustered together in the bootstrap test (1000 replicates) [37]. The outgroup was *Acaulospora spinosa*.
